# Topical treatment of equine aural plaques with nitric acid and zinc solution

**DOI:** 10.1002/evj.70161

**Published:** 2026-03-12

**Authors:** Lii Katarina Leo, Kerstin Elisabet Bergvall

**Affiliations:** ^1^ Department of Clinical Sciences, Equine Clinic University Animal Hospital Swedish University of Agricultural Sciences Uppsala Sweden

**Keywords:** aural plaques, horse, papilloma virus, topical treatment

## Abstract

**Background:**

Equine aural plaque caused by equine papilloma viruses is common worldwide and affected horses can show severe clinical signs. Due to its viral aetiology, in some countries affected horses are not allowed to compete. Since lesions persist, showing no spontaneous regression, effective and safe treatment is required. Currently, the only treatment with consistent efficacy is imiquimod. Treatment time is prolonged and adverse effects are common. In humans, papilloma warts are successfully and safely treated with topical nitric‐zinc complex solutions inducing a painless caustic effect.

**Objectives:**

To retrospectively evaluate the effectiveness and adverse effects of topical treatment with a nitric acid and zinc solution (Oxalic® or Verrutop®).

**Study Design:**

Retrospective case series.

**Methods:**

Records from horses with aural plaque diagnosed at the Equine Clinic of the University Animal Hospital and Equine Clinic, Täby, Sweden during 2012–2022 were reviewed. Included horses were treated topically with nitric acid and zinc solution (Oxalic® or Verrutop®), unilaterally or bilaterally every other week until remission. A successful outcome was defined as no remaining lesions at 12‐ to 28‐month follow‐up.

**Results:**

Twelve horses were included, eight bilaterally affected. In total 20 ears were treated. Complete remission was seen in 17 ears (1–5 treatments, median 1.5). The three remaining ears substantially improved and clinical signs of discomfort were no longer observed. No adverse effects were seen.

**Main Limitations:**

Retrospective study, small sample size, not blinded and no control group included. Lesions were diagnosed by macroscopic appearance; no PCR for virus detection or histology was made.

**Conclusion:**

Topical nitric–zinc complex solutions are a safe and effective treatment for equine aural plaques.

## 1 INTRODUCTION

Equine aural plaques, a papilloma virus associated condition, are common. Prevalences reported from Brazil range from 14.8% to 41% occurring in at least one horse on every yard surveyed.^1^, ^2^ Aural plaques typically begin as raised, small, depigmented papules on the concave surface of the pinna, usually developing into white hyperkeratotic plaque 1–3 cm in diameter.^3^ The lesions can be single or multiple and coalescing, unilateral or bilateral in affected horses and can progress to even covering the whole surface of the concave pinna. Aural plaques can be asymptomatic,^3^ but head shyness, difficulty bridling and sensitivity when touching the head or ears are common problems in affected horses.^4^, ^5^ There is a need for an efficacious and safe treatment option.

The lesions are caused by equine papillomavirus (EpV), and all types 1, 3, 4, 5 and 6 have been demonstrated in aural plaques.^2^, ^5^, ^6^, ^7^ Co‐infection with more than one type of the virus is common.^2^, ^7^ The virus is probably transmitted both by direct and indirect contact, needing biting flies and damage in the skin to be able to cause an infection. EpV 1 is also associated with viral papillomatosis affecting predominantly the muzzle with wart development in young horses. This is a condition that usually regresses spontaneously within 1–9 months, and treatment is often not necessary.^5^ Unlike viral papillomatosis, equine aural plaques do not seem to regress spontaneously. EpV 2 is associated with genital papillomatosis and is also involved in the pathogenesis of genital and ocular squamous cell carcinoma (SCC).^8^ EpV 8 has been detected in horses with severe, generalised papillomatosis^9^ and SCC.^10^ So far, EpV 2, 7 and 8 have not been detected in aural plaque lesions.^6^ However, there is one case report of a horse with aural plaque that developed SCC on the inside of the pinna.^11^ This horse had characteristic histopathological changes for both aural plaques and SCC and was also positive on PCR for EpV 4. The potential progress to SCC represents another reason for an effective and safe treatment protocol.

Several different treatment options have been tried, but currently only treatment with imiquimod has shown consistent efficacy.^4^, ^12^ Horses treated with imiquimod develop side effects (severe inflammation, erosion, ulceration, crusting and pain) and most of the horses need sedation for every application of the cream. The reported treatment period needed for resolution is prolonged (1.5–8 and 0.5–5 months).^4^, ^12^ The aim of this study is to describe treatment with a solution containing nitric acid, potassium nitrate, zinc nitrate and binder (Oxalic® previously provided by N‐vet) or a solution containing nitric acid, zinc, copper and the organic acids acetic acid, oxalic acid and lactic acid (Verrutop® provided by ISDIN, Barcelona, Spain). Both the nitric acid and zinc complex solutions (Oxalic® and Verrutop®) induce a painless caustic effect when treating warts in humans. The solution which contained nitric acid, potassium nitrate, zinc nitrate and binder (Oxalic®) was, when it was available, used for the treatment of warts in companion animals, and the now available solution containing nitric acid, zinc, copper and the organic acids acetic acid, oxalic acid and lactic acid (Verrutop®) is used for treatment of warts caused by papilloma virus in humans where the treatment results do not differ significantly from cryotherapy. As the treatment is painless, it is considered as one of the first line treatment options for human papilloma warts.^13^ The treatment is also used when treating paediatric patients with satisfactory results.^14^ Our hypothesis is that treatment with nitric acid and zinc complex solutions (Oxalic® or Verrutop®) is effective and safe for treatment of aural plaques.

## 2 MATERIALS AND METHODS

**FIGURE 1:**
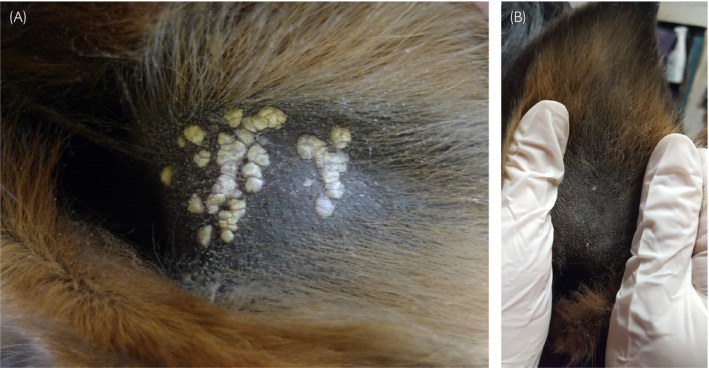
(A) The macroscopic appearance of a horse with equine aural plaque on the inside of the pinna. (B) The macroscopic appearance of the same horse as (A), at follow‐up after treatment with the nitric acid and zinc complex containing solution.

**FIGURE 2:**
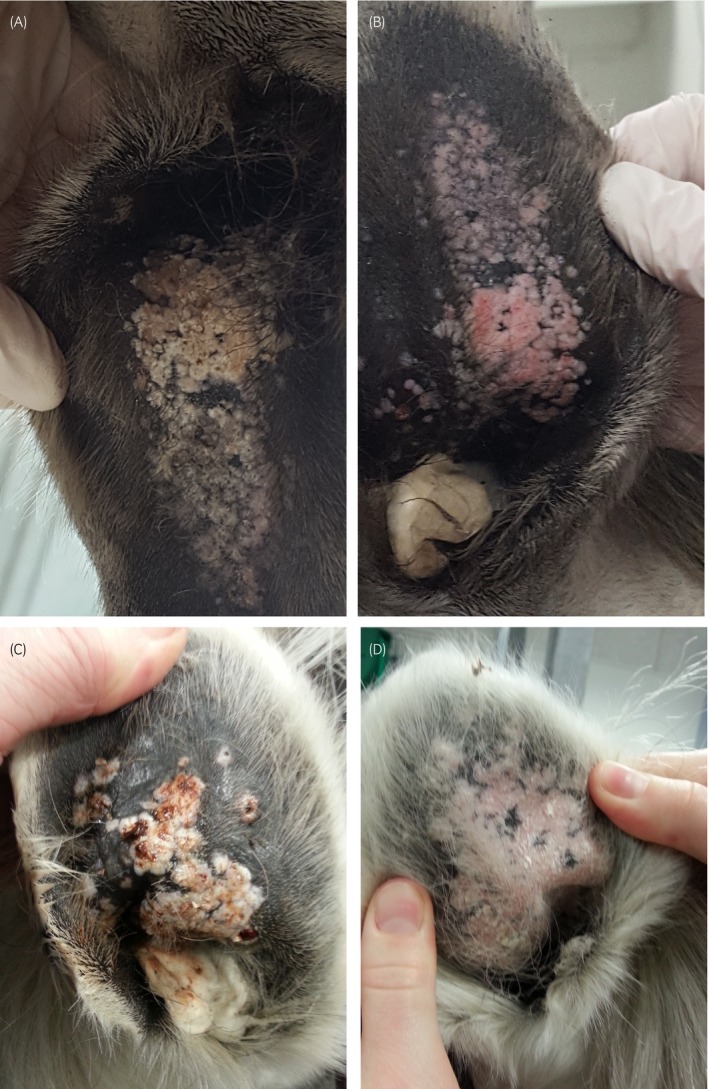
(A) Macroscopic appearance of equine aural plaque before treatment of the inner surface of the pinna. (B) The macroscopic appearance after cleaning and removing the waxy coating. Removing the waxy outer layer is important to allow the nitric acid and zinc complex solution to properly reach the tissue. Note the unrefined cotton wool protecting the ear canal from eventual liquid running down. (C) The macroscopic appearance immediately after applying the nitric acid and zinc complex solution. The lesions turn whitish after treatment application. (D) Follow‐up on the same horse as (A), (B) and (C). The picture shows the macroscopic appearance of the inner surface of the pinna two weeks after treatment with the nitric acid and zinc complex solution.

Medical records of horses diagnosed with aural plaque between 2012 and 2022 were reviewed. Horses that had typical macroscopic lesions on the concave aspect of the pinna, unilateral or bilateral (Figures 1A and 2A) and were treated with nitric acid and zinc complex solutions (Oxalic® or Verrutop®) were included in the study (Figure 1B). All the horses were examined by a Diplomate in veterinary dermatology.

The horses were, after physical examination, sedated with a combination of detomidine hydrochloride (Domosedan® vet. 10 mg/mL, Orion Pharma Animal Health) and butorphanol (Butomidor® vet. 10 mg/mL, Salfarm Scandinavia) intravenously. To protect the ear canal and the tympanic membrane during treatment, the ear was packed with unrefined cotton wool (Figure 2B). The hair on the inside of the pinna was then clipped, and the skin was cleansed with alcohol to remove the waxy coating over the lesions before application of the nitric acid and zinc complex solution (either Oxalic® or Verrutop®). The solution was applied with the help of a 2‐mL syringe until all the lesions had been covered; on average, 0.3 mL of solution was used per cm^2^ (Figure 2C). The treatment was repeated, if necessary, with a two‐week interval without any intervention by the owner at home. The minimum treatment period, even if the horse was only treated at one single occasion, was recorded as 2 weeks (until re‐check). If the horse needed more than one treatment, the treatment length was calculated from the first day to 2 weeks after the last application. The treatment was considered successful if all the lesions were healed on revisit and if the horse had improved clinically (less head‐shy, less sensitive to touch and easier to bridle) (Figure 2D). Any kind of side effects were recorded. At least 1 year after the last treatment, horses were either re‐examined at our clinic (four horses) or the owners (eight horses) were contacted by telephone and asked for information on whether the condition had recurred.

## 3 RESULTS

Cases are summarised in Table S1. Twelve horses were included in the study, eight were bilaterally affected and two had only the right or the left ear affected. In total, 20 ears were treated. Ten ears (50%) each were treated with either of the nitric acid and zinc complex solutions (Oxalic® or Verrutop®, Verrutop® replaced Oxalic® from 2020). The horses with bilaterally affected ears had both ears treated under the same sedation. Eight of the 12 horses were geldings and four were mares, the breeds included were Swedish warmblood (*n* = 3), crossbreed pony (*n* = 2), Pura Rasa Espanola (*n* = 1), Connemara (*n* = 1), Standardbred trotter (*n* = 1), Arabian thoroughbred (*n* = 1), Icelandic horse (*n* = 1), German warmblood (*n* = 1) and Belgian draught horse (*n* = 1). The age ranged from 3 to 15 years of age (median: 8.5 years). The owners of all horses reported prominent clinical signs, including head shyness, were sensitive to touch of the ears and had problems while bridling or putting a halter on (100%). The number of treatments per ear varied (median: 3 range 1–5). The horses were sedated before every treatment and the number of times horses were sedated therefore varied (median: 1.5, range 1–5). The duration of treatment varied between 2 weeks up to 21 weeks (median: 3.5 weeks). In total 17 of 20 ears (85%) went in complete remission after treatment in 10 of the 12 horses. In the remaining three ears (15%), the lesions were notably milder than before treatment and both these horses (one unilateral and one bilaterally affected horse) had improved clinically. Prior to treatment, touching the ears was not possible in any of the horses and to place bridles or halters, the equipment had to be dismantled to avoid getting close to the ears. After treatment, the owners reported that they had no problems handling the ears or bridling in the horses with complete remission and those in which the lesions were not completely resolved. Information was obtained for all 12 horses 12–28 months after treatment. Only one horse had relapsed in this time period. Of the two horses (three ears) that still had lesions following treatment, one horse (one ear) relapsed and clinical signs of discomfort developed 3 years after initial treatment, information we obtained because the owner contacted the clinic when the horse again showed signs of discomfort. The other horse (both ears) did not develop new signs of ear sensitivity for 4 years after treatment, information we obtained because the horse presented at our clinic for other reasons and was euthanised (due to limb fracture).

## 4 DISCUSSION

### 4.1 Outcome

The result from this study shows that 17 of the total 20 ears (85%) corresponding to 10 out of 12 horses (83%) went into complete remission after treatment. This is in line with the long‐term resolution from imiquimod studies.^4^, ^12^ Although, Zakia et al. report a higher success rate where 93% of the treated ears healed completely.^12^ However, four of the eight included horses in that study needed more than one treatment sequence for complete resolution. In our study, the two horses that still had active lesions after initial treatment, albeit markedly smaller than before, no longer exhibit clinical signs of discomfort as previously expressed (head shyness and sensitivity touching the ears). These two horses (one unilaterally and one bilaterally affected) were only treated two and three times, and additional treatment occasion/s could possibly have resulted in complete resolution but, since the horses did not show any discomfort, no more treatment was given.

### 4.2 Treatment period and number of treatment occasions

The number of treatments in this study varied between one to five. Several horses needed just one treatment (10/20 ears, 50%) for complete resolution of the lesions and therefore only needed to be sedated once. The number of treatments was substantially fewer compared to both previously published studies on imiquimod. In Torres et al., 18–96 treatments (mean: 36, median: 33 treatments),^4^ and in Zakia et al., 8–30 treatments (mean: 18, median: 18 treatments) before complete resolution of the lesions were reached.^12^ All the horses in our study needed sedation to be able to manage to apply the treatment; the total number of sedations was therefore the same as treatment occasions. The horse that needed five treatments was subsequently sedated on five different occasions. In the studies with imiquimod, due to the horses' original clinical signs and the inflammation caused by the treatment, 63% needed sedation before each application with imiquimod (range: 18–96 treatments),^4^ and in the study by Zakia et al. the number of sedations varied from 0 to 29, with a mean of 17.^12^ The difficulty cleaning the ears before application of imiquimod caused discomfort during treatment, and the need for sedation were the main concerns expressed by the owners in these studies.^4^ The treatments with nitric acid and zinc complex solution (Oxalic® or Verrutop®) were done by a veterinarian; the owner did not need to clean the ear or apply anything at home between treatments and therefore owner compliance has minimal impact. Torres et al. considered the ability to clean the ears before imiquimod treatment affected the treatment length, and owner compliance is therefore very important.^4^ In our study, the treatments were performed in a clinic but, since no special equipment is needed, the treatment is suitable also for field conditions.

The treatment length is very varied with imiquimod, ranging from 2 to 20 weeks in one study,^12^ and 6 to 48 weeks in the other published study.^4^ The difference in length may depend on the frequency of treatment application, which differed between these two studies. In our study, the treatment length varies between 2 and 21 weeks, with a maximum of five treatments. Many (10/20 treated ears) needed only one treatment application, which correlates to a treatment period of only 2 weeks. A few of the horses included had longer than the desired 2 weeks between treatment occasions, which made the treatment duration reported in this study longer than it would need to be according to treatment numbers (maximum five treatments).

### 4.3 Adverse effects

All the treated horses had obvious clinical signs of discomfort before treatment that gradually decreased during the treatment period. None of the horses treated with the nitric acid and zinc complex solutions (Verrutop® or Oxalic®) developed any side effects and the solutions used in our study did not induce any obvious inflammation. In contrast, horses treated with imiquimod all showed adverse effects to treatment (inflammation, oedema, erosions and ulcerations).^4^, ^12^ Some horses in the studies with imiquimod developed severe adverse effects to the degree where the owners could not complete the treatment course. The horses treated with the nitric acid and zinc complex solutions (Verrutop® or Oxalic®) showed decreased discomfort from the ears during the treatment period, with the ears being easier and easier to palpate and inspect prior to sedation and next treatment and the owners reported less severe discomfort at home even before the lesions were completely healed. When the nitric acid and zinc complex solution (Verrutop®) is used on dogs with nodular sebaceous gland hyperplasia, none of the dogs included needed sedation or anaesthesia during treatment, which indicates that applying the solution is probably not painful.^15^ The lesions initially white, slightly pink after cleaning with alcohol, usually turn distinctly whitish again after application, which makes it easy to see if all the lesions have been treated. Eventually, remaining lesions that needed treatment on follow‐up examination are therefore easy to distinguish.

### 4.4 Relapse

In total, three horses relapsed in the current study, of which only one relapsed within our follow‐up period of 12–28 months. In one imiquimod study, four of the included horses relapsed after the first round of treatment, two of these four horses relapsed again after the second round of treatment and needed a third round of treatment.^12^ During the 6‐month follow‐up after complete resolution of the aural plaque in that study, no horse relapsed. In another imiquimod study, 2 of the 16 horses that completed the treatment with complete remission relapsed during the 12‐ to 22‐month follow‐up period.^4^

The relapses in our study could be due to leaving existing lesions during treatment, or insufficient clearance of the virus. In our study no samples for virus detection were taken. Zakia et al. reported virus was still detectable after treatment in a few horses with no obvious macroscopic lesions remaining.^12^ Another possibility could be reinfection with the virus. The horse in our study that relapsed after 2 years needed one more treatment with the nitric acid and zinc complex solution (Verrutop®) to resolve the new lesions. The horse that relapsed after 3 years did so after being diagnosed with anaplasmosis and the third horse relapsing at 4 years was severely affected bilaterally the first time and also had the longest treatment period with the most treatment occasions initially (five). There is a possibility that this horse had insufficient virus clearance during the first treatment period. The horse was otherwise healthy and showed no other clinical signs during the treatment period. The horse was again treated with the nitric acid and zinc complex solution (Verrutop®) bilaterally and responded well.

## 5 CONCLUSIONS

Treatment for aural plaque with the solution containing nitric acid, potassium nitrate, zinc nitrate and binder (Oxalic®) or a solution containing nitric acid, zinc, copper and the organic acids acetic acid, oxalic acid and lactic acid (Verrutop®) was as effective as treatment with imiquimod but required fewer treatments under sedation and there were no side effects. Adverse effects are common with imiquimod, some so severe that the treatment had to be prematurely discontinued. The total treatment time until cure was shorter and relapse rate was low in the current study and similar to treatment with imiquimod. Owners do not need to intervene between treatments, and the treatment can also be performed in the field. In conclusion, this treatment protocol is cheap, safe, effective and causes less pain for the horse.

## FUNDING INFORMATION

Not applicable.

## CONFLICT OF INTEREST STATEMENT

The authors declare no conflicts of interest.

## AUTHOR CONTRIBUTIONS

**Lii Katarina Leo:** Writing – original draft; writing – review and editing. **Kerstin Elisabet Bergvall:** Methodology; writing – review and editing; writing – original draft; supervision.

## DATA INTEGRITY STATEMENT

Lii Leo had full access to all the data in the study and takes responsibility for the integrity of the data and the accuracy of data analysis.

## ETHICAL ANIMAL RESEARCH

Research ethics committee oversight not required by this journal: retrospective study of clinical records.

## INFORMED CONSENT

Not applicable.

## Supporting information


**Table S1:** Overview over the treated horses, with ear affected, number of treatments and outcome.

## Data Availability

The data that support the findings of this study are available upon reasonable request from the corresponding author. Open data sharing exemption granted by the editor.
