# Supplementary material for: Topical treatment of equine aural plaques with nitric acid and zinc solution
Source: Equine Vet J. 2026 Mar 12;58(5):1252–7. doi: 10.1002/evj.70161 (PMC13447932; doi:10.1002/evj.70161)
Supplement: Supplementary file 1 [file EVJ-58-1252-s001.pdf]

Equine Veterinary Journal BEVA

Table S1: Overview over the treated horses, with ear affected, number of treatments and outcome. Ears affected: R=Right; L=Left. Outcome: CR=Complete remission; PR=Partially remission; (R)=Relapsed during the follow-up period (12-28 months).

Horse	Ear affected	Number of treatments	Outcome
1	R	1	CR
2	R	2	CR
2	L	3	CR
3	R	1	CR
3	L	2	CR
4	L	3	PR
5	R	1	CR (R)
5	L	1	CR (R)
6	R	2	PR
6	L	2	PR
7	R	1	CR
7	L	1	CR
8	R	5	CR
8	L	4	CR
9	R	3	CR
9	L	3	CR
10	R	1	CR
10	L	1	CR
11	R	1	CR
12	L	1	CR
